# DNA repair/replication transcripts are down regulated in patients with Fragile X Syndrome

**DOI:** 10.1186/1756-0500-6-90

**Published:** 2013-03-11

**Authors:** Huichun Xu, Mónica A Rosales-Reynoso, Patricio Barros-Núñez, Emmanuel Peprah

**Affiliations:** 1 Center for Research on Genomics and Global Health, National Human Genome Research Institute, National Institutes of Health, 12 South Dr. MSC 5635, Bethesda, MD 20892, USA; 2 Centro de Investigación Biomédica de Occidente, Instituto Mexicano del Seguo Social, Sierra Mojada 800. Col. Independencia, Guadalajara, Jalisco, 44340, Mexico

**Keywords:** Haploinsufficiency, FMR1, DNA repair/replication proteins

## Abstract

**Background:**

Fragile X Syndrome (FXS) and its associated disorders are caused by the expansion of the CGG repeat in the 5’ untranslated region of the fragile X mental retardation 1 (*FMR1*) gene, with disease classification based on the number of CGG repeats. The mechanisms of repeat expansion are dependent on the presence of *cis* elements and the absence of *trans* factors both of which are not mutually exclusive and contribute to repeat instability. Expansions associated with *trans* factors are due to the haploinsuffient or reduced expression of several DNA repair/metabolizing proteins. The reduction of expression in *trans* factors has been primarily conducted in animal models without substantial examination of many of these expansion mechanisms and *trans* factors in humans.

**Results:**

To understand the *trans* factors and pathways associated with trinucleotide repeat expansion we have analyzed two microarray datasets which characterized the transcript expression in patients with FXS and in controls.

**Conclusion:**

We observed significant down regulation of DNA damage/repair pathway transcripts. This observation was consistent in both datasets, which used different populations. Within these datasets, several transcripts overlapped in the direction of association and fold change. Further characterization of these genes will be critical to understand their role in trinucleotide repeat instability in FXS.

## Background

Fragile X Syndrome (FXS, OMIM 300624) is caused by a mutation in fragile X mental retardation 1 (*FMR1*). Prevalence estimates for FXS in the general Caucasian population is ~1 out of 4000 males and ~1 out of 8000 females [1,2]. This prevalence rate has been subsequently substantiated by other reports, thus the rate is generally regarded as the prevalence rate in a randomly mating population [3]. FXS is a spectrum disorder in which affected individuals have IQs ranging from low or moderate to high functioning [4,5]. In over 98% of patients, FXS is caused by expansion of the CGG repeats in the 5’ untranslated region of *FMR1* located adjacent to exon1 on the X chromosome [6,7]. The CGG repeat region can be grouped into four general allelic forms, based on the CGG repeat length and stability, during transmission from parent to child. The allelic forms include common variants containing 6–40 repeats; intermediate variants, sometimes termed gray zone alleles, containing 41–54 repeats; premutation variants containing 55–199 repeats; and the full mutation variants, containing >200 repeats [1]. When the expansion exceeds 200 CGG repeats (i.e. full mutation), it causes methylation of the *FMR1* regulatory region, which induces transcriptional silencing [8]. This CGG repeats are periodically interspersed with AGG interruptions; interestingly the presence of these AGG interruptions in the CGG repeat have been shown to reduced the risk of transmission of a full mutation to offspring [9]. The transmission of the FXS full mutation is usually maternally derived because sperm from males with a premutation or a full mutation only carry premutation alleles; however one case of a premutation male who transmitted a full mutation to his daughter is reported in the literature [10].

Similar to other trinucleotide expansion disorders (e.g., myotonic dystrophy, Huntington disease, and some spinocerebellar ataxias) FXS CGG repeat expansions are locus specific; that is, genome-wide instability is not observed. This suggests that the mechanism of repeat expansions might not be caused by mutations in the DNA repair proteins or other *trans* acting factors, as such factors typically lead to genome-wide instability (e.g., several colorectal cancers and diseases [11]).

Other mechanisms have been proposed to produce trinucleotide repeat expansions that do not require a mutation phenotype [12]. One mechanism suggests the alternation of the stoichiometric amount of protein product needed to maintain genomic integrity could underlie trinucleotide repeat expansions without the mutation phenotype. For example, a reduction in the stoichiometric ratio of enzymes critical for DNA repair/replication leading to locus-specific expansions has been investigated in animal models [13,14]. Haploinsufficiency has mainly focused on the proteins in the ATM/ATR pathway to understand the intergenerational expansions of CGG repeats [13,14]. Reports have clearly demonstrated that haploinsufficiency of ATR and ATM (kinases that function in resolution of stalled replication forks and in double strand breaks, respectively) leads to increased intergenerational expansion of CGG repeats with a maternal and paternal bias, respectively [13,14]. Corroborating evidence of haploinsufficient expression of DNA repair/replication protein transcripts has not been reported in humans. Recently, expression analysis of transcripts has occurred in patients with FXS [15-18]. We used two datasets to determine if expression transcripts of DNA repair/replication enzymes were significantly altered in patients with FXS compared to controls. We observed that within both datasets there was significant down regulation of several enzymes with characterized functions in DNA repair and replication. In addition, both datasets also showed an overlap of several proteins important in maintaining genomic integrity.

## Methods

Genes in DNA repair/replication pathways were compiled from the REACTOME pathway database (http://www.reactome.org). FXS expression array data were obtained from two sources. First, microarray expression dataset GSE7329 was downloaded from the National Center for Biotechnology Information Gene Expression Omnibus (http://www.ncbi.nlm.nih.gov/geo/) database [16]. In this dataset, expression profiles of blood-derived lymphoblastoid cells from males with autism due to a fragile X mutation (FMR1-FM), or autism due to a 15q11-q13 duplication (dup(15q)), or individuals without autism spectrum disorders (i.e. controls), were studied using Agilent Whole Human Genome Oligo Microarray G4112A. Probes corresponding to the compiled list of genes in DNA repair/replication pathways were identified on the Agilent Whole Human Genome Array. Log (base 10) transformed expression values were extracted from the downloaded GSE7329 dataset directly. Analysis of Variance (ANOVA), followed with specific contrast between FXS and control groups, was conducted in Partek Genomics Suite 6.5 (Partek, Inc., St. Louis, Missouri, USA). Second, gene expression related to DNA repair/replication pathways were also assessed using data from our collaborators. Recently, Rosales-Reynoso and colleagues published a gene expression profiling study of the total peripheral blood from 10 male patients with FXS and 10 controls [15]. Two-color Human Genome Microarray (MWG Biotech H10K_DB) from the Physiology Laboratory of the Universidad Nacional Autónoma de México (http://microarrays.ifc.unam.mx) was used for this study. Target preparation, hybridization, and initial data collection were performed according to the Physiology Laboratory’s in-house protocol. Signal quantification and normalization were determined using the Array-Pro Analyzer 4.0 software for microarray images (Media Cybernetics, L.P., Silver Spring, MD). Details about the experiment protocol have been described previously [15]. Probes corresponding to the compiled list of genes in DNA repair/replication pathways were extracted from the dataset. One sample *t*-test was conducted for the Log transformed expression intensity ratios between pairs of patients with FXS and controls in Partek Genomics Suite 6.5. False discovery rate control (FDR < 0.05) was applied to indentify the differentially regulated genes.

## Results

**Figure 1:**
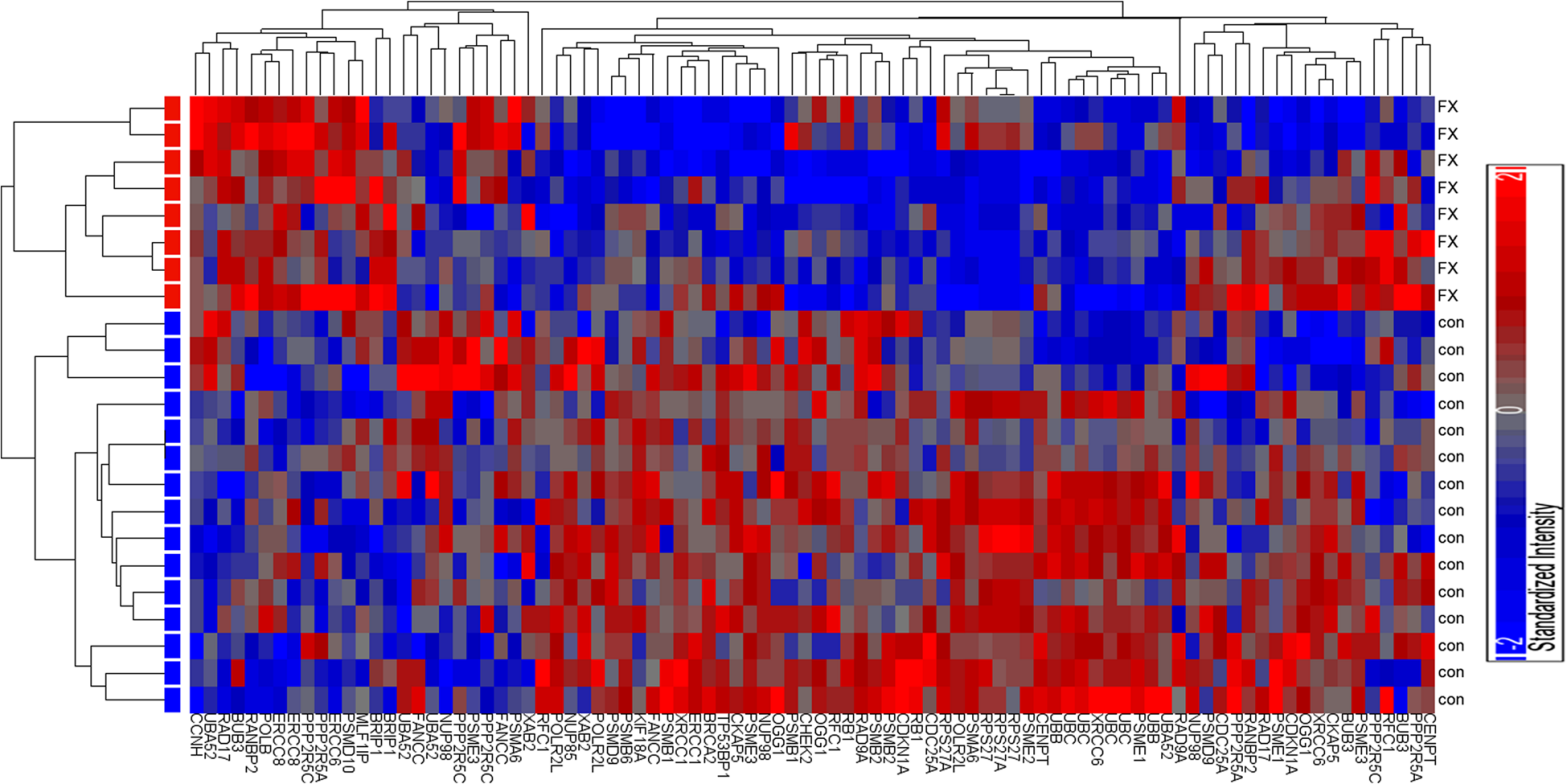
**Cluster heatmap showing expression profiles of DNA repair**/**replication related genes in individuals with fragile X Syndrome (FX) and control subjects (con).** Individual transcripts are shown on the x-axis and individual subjects are shown on the y-axis. The gene expression intensities are color-coded with red being high and blue being low, as indicated by the color scale to the right. Fifty-six transcripts from 45 genes were used for an unsupervised Pearson cluster analysis. These 45 genes demonstrated differential regulation between patients with FXS and control subjects according to ANOVA analysis with FDR < 0.05. Similarities between subjects or transcripts are represented by the distances between branches to the left and on the top, respectively. The closer the branches, the more similar the expression profiles of the corresponding subjects/transcripts.

Rosales-Reynoso and colleagues reported significant down regulation of Rad9A transcript, a DNA repair/cell cycle check point protein within the ATR/ATM DNA repair pathway, and up regulation of MSH6 (DNA mismatch repair binding protein) in patients with FXS [15]. A decrease in Rad9A transcripts could significantly impair DNA repair/replication pathway which could lead to repeat expansion. To further investigate the haploinsufficiency hypothesis, we examined the microarray datasets looking specifically at genes in DNA repair/replication pathways according to the curated REACTOME pathway database (http://www.reactome.org). A total of 312 genes involved in DNA repair and replication were obtained from REACTOME. Among them, 277 genes have corresponding probes (522 probes) covered by the Agilent Whole Human Genome Array G4112A which was used in the GEO dataset. Analysis of Variance (ANOVA) shows that 97 genes (35.0%) were differentially regulated between FXS samples and controls. Interestingly, the majority of the 97 genes (63 genes, 64.9%) were significantly down regulated (*p* < 0.05). After correction for multiple testing, 45 genes (46.4%) passed the false discovery rate threshold (FDR < 0.05). The majority of these genes (33 out of 45 genes, 73.3%) were down regulated (Figure 1). Expression data for several transcripts are shown in Figure 1, including down regulated ATM, Rad9A, and OGG1 genes that are implicated in trinucleotide expansion [12,13]. To corroborate these findings, we used the dataset from a Mexican population with FXS [15]. This dataset contained 175 unique genes represented on the arrays by 364 probes. Similar to the GEO dataset, the majority of genes in the DNA repair/replication pathway were down regulated. 109 genes were differentially regulated between patients with FXS and controls (*p* < 0.05). 92 genes passed the false discovery rate threshold (FDR < 0.05). Of the genes that passed the false discovery rate threshold, 89 were down regulated with 60 genes having greater than 1.5-fold changes. We compared the overlap of genes between the two datasets and found that 16 genes were present within both datasets which had similar fold change (Table 1).

**Table 1:** **DNA damage**/**repair genes which overlap in both datasets with similar fold**-**change and direction of association**

**Gene symbol**	**p**-**value**	**FDR corrected p**-**value**	**Fold change(FX vs. con)**	**Fold**-**change direction(FX vs. con)**
BRCA2	0.004	0.01	−1.17	down
CDKN1A	0.02	0.03	−1.11	down
ERCC1	0.0003	0.04	−1.06	down
FANCC	0.02	0.001	−1.08	down
NUP98	0.002	0.01	−1.10	down
PSMB1	0.02	0.02	−1.04	down
PSMD9	0.0004	0.03	−1.08	down
PSME2	0.0001	0.008	−1.07	down
PSME3	0.0005	0.003	−1.10	down
RFC1	0.003	0.02	−1.06	down
BUB3	0.02	0.009	1.10	up
POLB	0.007	0.01	1.08	up
PPP2R5A	0.02	0.02	1.05	up
PSMD10	0.005	0.01	1.03	up
RAD17	0.01	0.01	1.05	up
RANBP2	0.03	0.05	1.04	up

## Discussion

**Figure 2:**
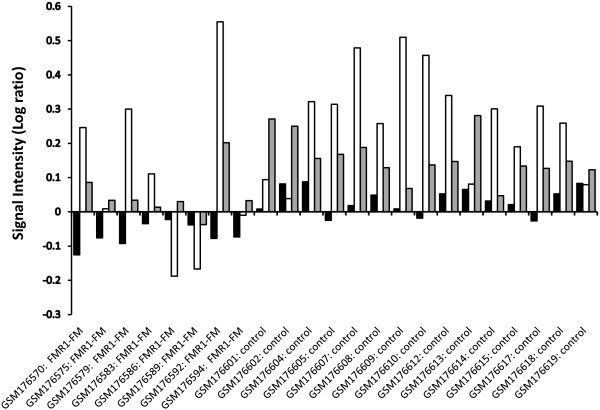
**Signal intensity Log ratios.** Differential expression of Rad9A (black bars), ATM (white bars), and OGG1 (gray bars) in cell lines containing FMR1 full mutation (FM) and controls. Rad9A and OGG1 showed decreased expression of transcript in FMR1-FM cell lines compared to controls. In a few FMR1-FM cell lines we observed a significant decrease in ATM (GSM176575, GSM176586, and GSM176589); however, the expression of ATM was not significantly different from FMR1-FM and controls.

Taken together, these data suggest differential expression of transcripts in the DNA repair/replication in patients with FXS compared to controls. Within patients with FXS there is considerable heterogeneity in the expression of many of the DNA repair/replication proteins (Figure 2). This could suggest that differential expression of several transcripts could lead to repeat expansion in some families essentially these families could be more susceptible to gene specific repeat expansions [19]. The down regulated transcripts could also suggest that there are several different pathways or combinations of down regulated transcripts which could lead to repeat expansion. The outcome of CGG expansion could occur through different mechanisms within individuals as suggested by animal models [13]. In fact, it is suggested that females transmit large expansions which appear to be repair dependent whereas males selectively delete large expansion and transmit small expansions which are replication dependent [12]. This observation is supported by haploinsufficient animal models [12,13]. Based on animal models and human data, we would suggest first characterizing the CGG expansion within the mother [if possible], with subsequent analysis in offspring by stratification of family members with premutations or FXS based on gender (e.g. paternal or maternal origin). In essence this method, previously used by Nolin and colleagues would allow for multi-generational characterization of the repair/replication pathways essential for expansion [19].

Transcript expression data may not have a linear association with translation; subsequent in vitro follow-up is needed to determine whether differential expression of transcripts in the DNA repair/replication pathway corresponds to alteration of protein levels in these enzymes. Further investigations are needed to provide models which couple decreased expression of DNA repair/replication enzymes with the mechanism of trinucleotide repeat expansion in patients with FXS. Other evidence from mouse models also shows a maternal and paternal bias in the inheritance of expanded repeats based on specific DNA replication/repair enzyme [12,13]. In addition, familial studies of intergeneration expansion have indicated evidence of different expansion rates [18]. This suggests that characterizing the mode of inheritance of the CGG expansion (i.e., whether primarily maternal or paternal) would be significant in understanding the inheritance. In effect, the mode of inheritance would provide significant data leading to an understanding of the enzymes which are significantly associated with expansion pathways.

Currently, only around 16 genes were found to be differentially regulated with the direction of association similar in both datasets. The limited number of genes found through this analysis is due to the fact that two different microarray experiments did not have a large number of overlapping genes for the DNA damage/repair pathway from REACTOME. We found that the degree of overlap between both datasets was around 50%. However, within this limited amount of data we found significant associations which we hope can be replicated by others. Overall, these findings indicate that the DNA damage/repair pathways could significantly contribute to repeat instability.

The current understanding of trinucleotide expansion disorders suggests that many of these expansions arise from several different mechanisms [12]. A second level of complexity would also suggest that the DNA repair/replication mechanisms could have considerable cross talk [12]. Understanding the mechanism of trinucleotide repeat expansion in FXS would be beneficial to understanding other trinucleotide repeat expansion disorders. Finally, the evolutionary significance of loci-specific repeat expansion disorders should not be understated. Evidence from this field could engender greater understanding of the evolution of the human genome and a greater understanding of how genome fidelity is maintained.

## Abbreviations

ATR: Ataxia telangiectasia and Rad3 related; ATM: Ataxia telangiectasia mutated; ANOVA: Analysis of Variance; FDR: False discovery rate; FMR1: Fragile X mental retardation 1 gene; FXS: Fragile X Syndrome; GEO: Gene Expression Omnibus; NCBI: National Center for Biotechnology Information; OGG1: 8-oxoguanine DNA glycosylase; OMIM: Online Mendelian Inheritance in Man; Rad9A: DNA repair exonuclease rad9 homolog A

## Competing interests

The authors declare that they have no competing interests.

## Authors’ contributions

HX, analyzed data for both data sets and contributed to the manuscript, MRR and PBN prepared and analyzed samples for the Mexican populations, and EP conceived experiments and wrote the manuscript. All authors read and approved the final manuscript.

## Datasets

Dataset for Mexican population was obtained from Dr. Patricio Barros-Núñez (http://pbarros_gdl@yahoo.com) [15]. Dataset GSE7329 was downloaded from the National Center for Biotechnology Information Gene Expression Omnibus (http://www.ncbi.nlm.nih.gov/geo/) database [16].
